# Serum Levels and Placental Expression of NGAL in Gestational Diabetes Mellitus

**DOI:** 10.1155/2020/8760563

**Published:** 2020-01-03

**Authors:** Xiaoqian Yin, Yan Huo, Li Liu, Yixing Pan, Suxin Liu, Runfang Wang

**Affiliations:** Department of Obstetrics & Gynecology, Hebei General Hospital, Shijiazhuang 050051, China

## Abstract

**Objectives:**

The aim was to investigate neutrophil gelatinase-associated lipocalin (NGAL) levels in the serum and term placentas and its potential role in gestational diabetes mellitus (GDM).

**Methods:**

A total of 49 GDM subjects and 39 age-matched women with normal pregnancies were recruited. We examined serum concentrations of NGAL and tumor necrosis factor-*α* (TNF-*α*) in maternal blood and cord blood and their expression levels in the term placentas and umbilical cord.

**Results:**

Serum NGAL levels were significantly higher in GDM patients than in normal pregnant controls both in the maternal blood (4.80 ± 1.99 vs. 3.66 ± 1.13, *P*=0.001) and the cord blood (4.70 ± 2.08 vs. 3.85 ± 1.44, *P*=0.027). Moreover, serum NGAL levels exhibited a positive correlation with various parameters of insulin resistance. Maternal serum NGAL levels positively correlated with the NGAL levels found in the cord blood of the control (*r* = 0.399, *P*=0.012) and the GDM subjects (*r* = 0.349, *P*=0.014). Finally, the expression of NGAL protein levels in the placenta (1.22 ± 0.39 vs. 0.65 ± 0.23, *P* < 0.001) and umbilical cord (0.65 ± 0.23 vs. 0.25 ± 0.10, *P* < 0.001) were higher in GDM women than those noted in the control subjects. In the GDM group, maternal serum NGAL levels exhibited a positive correlation with placental NGAL mRNA and protein levels (*r* = 0.848, *P*=0.008; *r* = 0.636, *P*=0.011, respectively).

**Conclusions:**

NGAL may be an important adipokine involved in GDM and fetal development. The oversecretion of NGAL from the placenta may contribute to the elevated levels of serum NGAL in gestational diabetes mellitus.

## 1. Introduction

Gestational diabetes mellitus (GDM) is defined as any degree of carbohydrate intolerance with an onset during pregnancy [1, 2]. It has been shown that 7% of pregnant women experience complications with regard to the incidence of diabetes and that approximately 86% of these cases are women with GDM [2]. The pathogenesis of GDM remains unknown, although insulin resistance plays a fundamental role in its development. Increased insulin resistance in pregnant women and the loss of the function of the islet B cells are two major causes that result in impaired insulin secretion in GDM patients. Recently, it was recognized that adipokines, such as leptin, adiponectin, and visfatin, contributed to insulin resistance and metabolic diseases in GDM [3, 4].

Neutrophil gelatinase-associated lipocalin (NGAL), also known as lipocalin-2, is a new type of adipocytokine that acts as an inflammatory factor [5]. This molecule belongs to the member of the lipocalin superfamily. Recent studies have shown that the levels of NGAL are closely associated with the development of hyperglycemia and insulin resistance [6–8]. Previous clinical studies have demonstrated that the serum NGAL levels of patients with type 2 diabetes mellitus (T2DM) were significantly increased and that they exhibited a positive correlation with several indices of insulin resistance, such as fasting blood glucose [9, 10]. This suggested that NGAL played a biological role in addition to its involvement in diabetes mellitus. Furthermore, certain studies have found that serum NGAL levels in GDM patients were significantly higher than those in normal pregnant women and that the expression levels of NGAL in adipose tissues were significantly higher in GDM overweight women than in women with normal weight [11, 12]. A previous study has shown that the expression levels of NGAL protein in the placental tissues of patients with preeclampsia were higher than those in control subjects, suggesting that NGAL in the placenta may exert a compensatory response to the development of preeclampsia [13]. However, to the best of our knowledge, no study has investigated the NGAL expression in the term placenta of GDM.

To elucidate the potential role of NGAL in the pathogenesis of GDM and the development of the fetus, we examined its plasma levels in maternal blood and umbilical cord blood and its expression levels in placental and umbilical cord tissues in women with GDM and women with normal pregnancies. Furthermore, we investigated the correlation between NGAL and tumor necrosis factor-*α* (TNF-*α*), which has been proposed as a significant predictor of insulin resistance, affecting the insulin receptor signal transduction pathways [12, 14, 15]. In addition, TNF-α can induce the expression of NGAL in placenta [16].

## 2. Material and Methods

### 2.1. Subjects and Tissue Collection

The group consisted of 49 women with GDM aged 32.47 ± 4.68 and 39 age-matched (31.77 ± 4.96 years) pregnant women with normal glucose tolerance (NGT) test response that were admitted to the Department of Obstetrics and Gynecology of the Hebei General Hospital between December 2017 and March 2018. All pregnancies were singletons and required elective cesarean section owing to breech presentation and/or previous cesarean section. GDM was diagnosed according to the diagnostic criteria of the International Association of Diabetes and Pregnancy Study Groups (IADPSG) [2]. The criteria were as follows: any single threshold value of 75-gram, 2-hour oral glucose tolerance test (OGTT) that is met or exceeded (fasting value, 5.1 mmol/l; 1-hour value, 10.0 mmol/l; or 2-hour value, 8.5 mmol/l) between 24 and 28 weeks of gestation. All subjects were nonsmokers and were not receiving drugs that could affect carbohydrate metabolism. All GDM patients were treated by diet and exercise. Patients with pregnancy-induced hypertension, preeclampsia, premature rupture of membranes, or other pregnancy complications were excluded. All subjects were in good health, without any known disease. The present study was approved by the Human Ethics Committee of the Hebei General Hospital (2017, No. 174), and informed consents were obtained from all patients participating in the study protocol.

Blood samples were obtained from the maternal vein prior to the cesarean section and from the umbilical vein prior to the placenta delivery and following fetal delivery. Blood samples were collected in tubes without anticoagulants and immediately centrifuged (999 g, 10 min) after clotting. The serum was stored at −80°C until analysis. Placental tissues from the maternal side near the umbilical cord insertion site and umbilical tissues were obtained during cesarean sections within 10 min of delivery. The tissues were frozen in liquid nitrogen immediately and stored at −80°C until further analysis.

### 2.2. Laboratory Assays

The following biochemical markers were investigated: fasting plasma glucose (FPG), 1 h plasma glucose, and 2 h plasma glucose, following glucose loading (1 h PG, 2 h PG, 75 g 2 h OGTT); fasting plasma glucose in the third trimester, total cholesterol (TC), triglyceride (TG), high-density lipoprotein cholesterol (HDL-C), low-density lipoprotein cholesterol (LDL-C), and very low-density lipoprotein cholesterol (VLDL-C). These indices were analyzed using an automatic analyzer (Hitachi 7600-110, Japan) at the biochemical laboratory of the Hebei General Hospital. Fasting insulin (FINS) was measured using chemiluminescence assays (Cobas e 601, China). The insulin sensitivity was determined by the Homeostasis Model Assessment (HOMA) index according to the formula: HOMA-IR = FINS (*μ*U/ml) × FPG (mmol/l)/22.5.

Serum NGAL concentration was measured using Human NGAL/Lipocalin-2 ELISA Kit (Neobioscience, China). The detection limit was set at 40 pg/ml. Serum TNF-*α* level was quantified using Human TNF-*α* ELISA Kit (Neobioscience, China) and the detection limit was at 7.8 pg/ml. The intra- and interassay coefficients of variation were less than 10% and 9.5%, respectively.

### 2.3. Total RNA Extraction and RT-qPCR

Total RNA was extracted from the placental tissues and umbilical cord blood samples using Trizol (TianGen, China) according to the manufacturer's instructions. The cDNA was synthesized from 2 *μ*g of total RNA using FastQuant RT Kit (with gDNase) (TianGen, China) as described in the manufacturer's instructions. The quantitation of NGAL and TNF-*α* genes was performed using PowerUp™ SYBR® Green Master Mix (Thermo Fisher, USA) and an ABI PRISM 7500 sequence detector (Applied Biosystems, USA). The protocol conditions were as follows: denaturation at 50°C for 2 min and at 95°C for 10 min, followed by 40 cycles of 95°C for 15 s, 58°C for 15 s, and 72°C for 1 min. Glyceraldehyde-3-phosphate dehydrogenase (GAPDH) was used as the internal standard. The primer sequences that were used in the present study were as follows: NGAL (115 bp), 5-AAGACAAAGACCCGCAAAAGATG-3 and 5-GTCCTGATCCAGTAGTCACACTTC-3; TNF-*α* (360 bp), 5-ACACGCTCTTCTGCCTGCTG-3 and 5-TCTGGTAGGAGACGGCGATGC-3; GAPDH (101 bp), 5-CAAGAAGGTGGTGAAGCAGG-3 and 5-AGTGGGTGTCGCTGTTGAAG-3. All the primers were synthesized by Invitrogen.

### 2.4. Western Blot Analysis

Placental tissues and umbilical cord blood samples were homogenized in radioimmunoprecipitation assay (RIPA) buffer (Solarbio, China) according to the manufacturer's instructions. The protein samples were resolved by polyacrylamide gel electrophoresis on 10% sodium dodecyl sulfate (SDS)-polyacrylamide gels and electrotransferred to polyvinylidene fluoride (PVDF) membranes. The membranes were blocked in Tris-buffered saline with Tween (TBS-T) and 5% skimmed milk for 2 h at room temperature and incubated overnight at 4°C with anti-NGAL (1 : 1000, CST, USA) and anti-TNF-*α* monoclonal antibodies (1 : 1000, CST, USA). The membranes were washed four times for 8 min in blocking buffer (TBS-T) and incubated for 1 h at room temperature with secondary antibodies (Goat Anti-Rat IgG, 3030-05, Southern Biotech; Rabbit Anti-Goat IgG, 6160-05, Southern Biotech). The incubation was followed by three times of washing in blocking buffer (TBS-T) for 10 min each. The immune complexes were detected by using the enhanced Electro-Chemi-Luminescence (ECL) kit (Cwbiotech, China).

### 2.5. Statistical Analysis

The data were assessed by the Kolmogorov–Smirnov test in order to examine whether they follow a normal distribution. For the data that followed a normal distribution, the values were presented as mean ± SD, and one-way analysis of variance (ANOVA) was implemented for group comparison. For the data that exhibited nonnormal distribution, the values were reported as medians (25–75%), and the Mann–Whitney *U* test was used for dual comparisons. The relationship between variables was analyzed by Pearson's correlation coefficients or Spearman's correlation coefficients, depending on the distribution of the variables. *P* values lower than 0.05 (*P* < 0.05) were considered to indicate significant differences in the comparisons of the data. Statistical analyses were performed using the SPSS 17.0 statistical software package (Statistical Analysis System, Chicago, IL, USA, version 22.0 for Windows).

## 3. Results

### 3.1. Clinical and Demographic Characteristics of the Subjects

The characteristics of all participants are summarized in Table 1. The two groups of pregnant women exhibited similar values with regard to the parameters age, gestational age, parity, prepregnancy BMI, and current BMI. The women with GDM exhibited significantly higher FPG1 (5.00 ± 0.47 vs. 4.47 ± 0.34, *P* < 0.01), 1 h PG (10.07 ± 1.38 vs. 7.16 ± 1.39, *P* < 0.01), 2 h PG (8.04 ± 1.32 vs. 6.34 ± 0.89, *P* < 0.01), FPG2 (4.92 ± 0.61 vs. 4.50 ± 0.40, *P* < 0.01), FINS (12.81 ± 4.62 vs. 10.72 ± 3.46, *P*=0.023), HOMA-IR (2.80 ± 1.11 vs. 2.15 ± 0.73, *P*=0.003), and TG (3.30 ± 1.36 vs. 2.63 ± 0.75, *P*=0.006) than those of normal pregnancy subjects. In addition, control subjects indicated a higher HDL-C (1.89 ± 0.27 vs. 1.76 ± 0.30, *P*=0.041). No significant differences in the variables TC (*P*=0.058), LDL-C (*P*=0.136), VLDL-C (*P*=0.558) and birth weight (*P*=0.092) were noted between the two groups.

### 3.2. Serum NGAL and TNF-*α* Concentration Levels in Maternal Blood and Cord Blood

**Figure 1:**
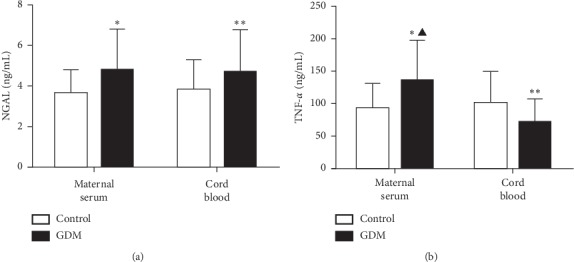
Comparison of serum NGAL and TNF-*α* concentrations in women with normal glucose tolerance (control) test response and subjects with gestational diabetes mellitus (GDM) in maternal blood and cord blood. (a) The bar chart indicates quantification of serum NGAL concentrations. The results are expressed as mean ± SD. ^*∗*^*P*=0.001: GDM compared with control in maternal blood; ^*∗∗*^*P* < 0.05: GDM compared with control in cord blood. (b) The bar chart indicates the quantification of serum TNF-*α* concentration levels. The results are expressed as mean ± SD. ^*∗*^*P* < 0.001: GDM compared with control in maternal blood; ^*∗∗*^*P*=0.001: GDM compared with control in cord blood; ^▲^*P* < 0.001: maternal blood compared with cord blood in GDM.

Fasting serum NGAL concentration levels were significantly higher in women with GDM than those in normal pregnancy subjects. These differences were evident both in maternal blood (4.80 ± 1.99 vs. 3.66 ± 1.13, *P*=0.001, Figure 1(a)) and in cord blood (4.70 ± 2.08 vs. 3.85 ± 1.44, *P*=0.027, Figure 1(a)). However, no significant differences were noted regarding serum NGAL between maternal blood and cord blood matrices both in the control subjects (*P*=0.522, Figure 1(a)) and the GDM participants (*P*=0.797, Figure 1(a)).

In addition, in maternal blood, the fasting serum TNF-*α* levels in the GDM group were significantly higher than those of the control group (136.55 ± 62.37 vs. 93.13 ± 38.87, *P* < 0.001, Figure 1(b)). In contrast to maternal blood, the TNF-*α* levels in cord blood were significantly lower in the GDM group than the corresponding levels in the control group (71.58 ± 36.43 vs. 102.44 ± 38.87, *P*=0.001, Figure 1(b)). Furthermore, in the group of GDM, the maternal blood levels of TNF-*α* were significantly higher than those in the cord blood for the subjects that experienced GDM (136.55 ± 62.37 vs. 71.58 ± 36.43, *P* < 0.001, Figure 1(b)).

### 3.3. Correlation between Serum NGAL Levels and Clinical and Demographic Characteristics

The correlations between maternal serum NGAL levels and clinical and demographic characteristics are shown in Table 2. In the control group, maternal NGAL levels correlated positively with fasting plasma glucose of the third trimester (*r* = 0.461, *P*=0.003), FINS (*r* = 0.358, *P*=0.025), and HOMA-IR (*r* = 0.479, *P*=0.002). In the GDM group, maternal serum NGAL levels correlated positively with the following parameters: fasting plasma glucose of the second trimester (*r* = 0.369, *P*=0.014), fasting plasma glucose at term (*r* = 0.390, *P*=0.008), FINS (*r* = 0.695, *P* < 0.001), HOMA-IR (*r* = 0.711, *P* < 0.001), and birth weight (*r* = 0.363, *P*=0.014). In addition, positive correlations were noted between maternal serum NGAL levels and fasting plasma glucose levels of the second trimester (*r* = 0.0.334, *P*=0.002), 1 h PG (*r* = 0.238, *P*=0.042), 2 h PG (*r* = 0.253, *P*=0.028), fasting plasma glucose levels of the third trimester (*r* = 0.486, *P* < 0.001), FINS (*r* = 0.559, *P* < 0.001), HOMA-IR (*r* = 0.624, *P* < 0.001), TG (*r* = 0.267, *P*=0.014), and birth weight (*r* = 0.350, *P*=0.001) in all the subjects included in the study.

Multiple linear regression analysis revealed a significant correlation with serum NGAL levels and FINS (beta = 3.066, *P*=0.001), HOMA-IR (beta = −2.679, *P*=0.007), fasting plasma glucose of the third trimester (beta = 1.133, *P*=0.002), and neonatal birth weight (beta = 0.219, *P*=0.028) in women with GDM.

### 3.4. NGAL and TNF-*α* mRNA Expression Levels in Placental and Umbilical Cord Tissues

**Figure 2:**
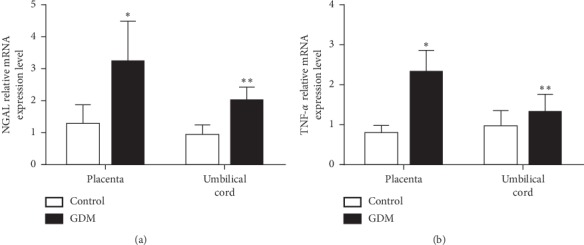
NGAL and TNF-*α* mRNA expression levels in placental tissues and umbilical cord blood samples of GDM and control groups. (a) Quantification of differences in NGAL mRNA expression levels between the two groups; ^*∗*^*P*=0.001, ^*∗∗*^*P* < 0.001. (b) Quantification of differences in TNF-*α* mRNA expression levels between the two groups; ^*∗*^*P*=0.001, ^*∗∗*^*P*=0.021. NGAL and TNF-*α* mRNA levels were corrected for the expression of the housekeeping gene, GAPDH.

The expression of NGAL mRNA levels in placental (3.24 ± 1.25 vs. 1.30 ± 0.57, *P*=0.001, Figure 2(a)) and umbilical cord (2.02 ± 0.41 vs. 0.96 ± 0.30, *P* < 0.001, Figure 2(a)) tissues were higher in GDM women than those in the control group. The expression levels of TNF-*α* mRNA in placental (2.56 (1.84, 2.70) vs 0.79 (0.61, 0.99), *P*=0.001, Figure 2(b)) and umbilical cord (1.16 (1.03, 1.64) vs 0.87 (0.72, 0.98), *P*=0.021, Figure 2(b)) tissues were also significantly higher in the GDM group than the corresponding levels of these parameters in the control group.

In the GDM group, the expression levels of NGAL mRNA in the placental tissues were significantly higher than those in the umbilical cord tissues (3.24 ± 1.25 vs. 2.02 ± 0.41, *P*=0.020, Figure 2(a)). Similar trends were noted for TNF-*α* mRNA levels (2.56 (1.84, 2.70) vs. 1.16 (1.03, 1.64), *P*=0.003, Figure 2(b)). However, no significant differences were noted in the expression levels of NGAL (*P*=0.189) and TNF-*α* (*P*=0.431) mRNA in placental and umbilical cord tissues in the control group.

### 3.5. NGAL and TNF-*α* Protein Expression Levels in Placental and Umbilical Cord Tissues

**Figure 3:**
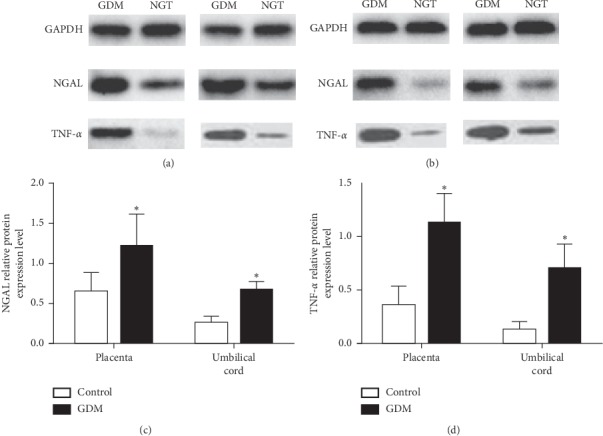
NGAL TNF-*α* protein expression levels in placental tissues and umbilical cord blood samples of the GDM and the control groups. (a) Representative western blot indicates the differences of NGAL and TNF-*α* protein expression levels in the placental tissues between the two groups; GAPDH served as an internal control. (b) Representative western blot indicates the differences of NGAL and TNF-*α* protein expression levels in umbilical cord samples between the two groups; GAPDH served as an internal control. (c) The bar chart indicates quantification of differences in NGAL protein expression levels among the two groups; ^*∗*^*P* < 0.001. (d) The bar chart indicates quantification of differences in TNF-*α* protein expression levels among the two groups; ^*∗*^*P* < 0.001.

NGAL protein levels were significantly higher in placental (1.22 ± 0.39 vs. 0.65 ± 0.23, *P* < 0.001, Figures 3(a) and 3(c)) and umbilical cord (0.67 ± 0.10 vs. 0.25 ± 0.10, *P* < 0.001, Figures 3(b) and 3(c)) tissues in the GDM group than the levels of NGAL in the control group. TNF-*α* protein expression levels in placental (1.09 (0.86, 1.37) vs. 0.28 (0.25, 0.57), *P* < 0.001, Figures 3(a) and 3(d)) and umbilical cord (0.69 (0.53, 0.87) vs. 0.11 (0.10, 0.16), *P* < 0.001, Figures 3(b) and 3(d)) tissues were significantly higher in GDM women than those in the control subjects.

The expression levels of NGAL protein in placental tissues were significantly higher than those in umbilical cord tissues (0.65 ± 0.23 vs. 0.25 ± 0.10, *P* < 0.001, Figures 3(a)–3(c)). Similarly, the TNF-*α* protein levels were significantly higher in placental tissues than those in umbilical cord tissues (0.28 (0.25, 0.57) vs. 0.11 (0.10, 0.16), *P* < 0.001, Figures 3(a), 3(b), and 3(d)). In the group of GDM, the protein levels of NGAL (1.22 ± 0.39 vs. 0.67 ± 0.10, *P* < 0.001, Figures 3(a)–3(c)) and TNF-*α* (1.09 (0.86, 1.37) vs. 0.69 (0.53, 0.87), *P* < 0.001, Figures 3(a), 3(b), and 3(d)) were markedly higher in placental than those in umbilical cord tissues.

### 3.6. Correlation between Serum NGAL Levels and Placental NGAL Expression

Furthermore, the correlations between maternal serum NGAL concentration levels, TNF-*α* and placental NGAL expression are shown in Table 3. Maternal NGAL levels positively correlated with cord blood NGAL (*r* = 0.399, *P*=0.012) in the control group. In the GDM group, maternal serum NGAL levels positively correlated with cord blood NGAL (*r* = 0.349, *P*=0.014), maternal serum TNF-*α* (*r* = 0.311, *P*=0.029), and placental NGAL mRNA and protein expression levels (*r* = 0.848, *P*=0.008 and *r* = 0.636, *P*=0.011). Moreover, maternal serum NGAL levels positively correlated with cord blood NGAL levels (*r* = 0.490, *P* < 0.001), maternal serum TNF-*α* levels (*r* = 0.248, *P*=0.020), and placental NGAL mRNA and protein expression levels (*r* = 0.740, *P*=0.001, *r* = 0.600, *P* < 0.001) in all the subjects included in the study.

## 4. Discussion

NGAL is a newly described adipocytokine, which is highly expressed in adipose tissues [17], and is associated with obesity, obesity-related inflammatory processes and insulin resistance. Mahfouz et al. [9] demonstrated that serum NGAL levels were significantly elevated in patients with T2DM, and that NGAL exhibited a positive correlation with insulin resistance status, specific insulin resistance markers (such as glycosylated hemoglobin and triacylglycerol) and diabetes progression. In 2009, D'Anna et al. [11] studied for the first time the association between NGAL and GDM and found that serum NGAL levels in GDM patients were significantly higher than those in women undergoing normal pregnancies and that they exhibited a positive correlation with the IR index. Lou et al. [12] reported significantly increased plasma NGAL levels in women with GDM, particularly among those with prepregnancy BMI over 25 kg/m^2^. Our results were consistent with these studies, since we found that serum NGAL levels, FINS, and HOMA-IR were significantly higher in women with GDM than those in women undergoing normal pregnancies. Moreover, we demonstrated that serum NGAL levels exhibited a positive correlation with fasting plasma glucose in the second trimester, fasting plasma glucose in the third trimester, and FINS and HOMA-IR in the GDM group. This implied that NGAL may be an indicator of disorders of glucose, lipid metabolism, and insulin resistance during pregnancy.

It still remains to be clarified how NGAL is linked to the deterioration of glucose metabolism and insulin sensitivity. NGAL can promote thermogenic activity via a norepinephrine (NE)-independent mechanism and can thus affect metabolic functions [18]. Chang et al. [19] have previously demonstrated that NGAL may impair *β* cell viability by promoting apoptosis in RINm5F *β* cells. The interaction of insulin with its receptor results in the phosphorylation of the insulin receptor substrate (IRS), which in turn allows the induction of expression of several downstream proteins, such as the proteins involved in the PI3K-Akt1 and the MAPK pathways. NGAL further caused insulin resistance via phosphorylation of Akt and p70S6K in cardiomyocytes (H9c2 cells), which was linked with the inhibition of autophagy [20]. In addition, numerous proinflammatory pathways promote insulin resistance. Thus, NGAL can block insulin signaling indirectly by promoting metabolic inflammation. TNF-*α* is a proinflammatory cytokine, which is involved in GDM and insulin resistance. Various reports have shown that TNF-*α* is significantly elevated in the serum of GDM patients compared with the serum of subjects undergoing normal pregnancies [21]. In the present study, TNF-*α* in GDM placenta was significantly increased compared with that in the control subjects. These results were consistent with Coughlan et al. [22], who showed that placental TNF-*α* release in vitro was significantly increased in GDM chorionic villi in comparison with ND under high glucose conditions. In contrast to Coughlan et al., Morei et al. [23] and Bari et al. [24] indicated that placental TNF-*α* release was not changed in GDM, although TNF-*α* release increased in placental tissues of pregnant women with DM2. These differences may be due to differences in the ethnicities and/or ages of the sample group, sample size, or GDM severity.

In this study, the serum NGAL and TNF-*α* levels were both increased in GDM women, whereas the serum NGAL levels exhibited a positive correlation with serum TNF-*α* levels. TNF-*α* can induce NGAL expression in the placenta [16]. Karlsen et al. [25] highlighted that TNF-*α* induces activation and binding of NF-*κ*B to the promoters of both NFKBIZ and NGAL genes, which in turn can induce transcription of I*κ*B-*ζ*. Costimulation with IL-17 enables synthesis of the I*κ*B-*ζ* protein, which binds to the NGAL promoter of NF-*κ*B and can induce NGAL expression. Therefore, the increased TNF-*α* induces the expression of NGAL in GDM.

The mechanism by which circulating NGAL levels were increased in GDM women at term may be associated with the adipose and placental tissues and with additional factors. Zhang and colleagues [26] demonstrated that insulin can stimulate Lcn2 expression and secretion in a dose-dependent manner by investigating the metabolic regulation of NGAL production in adipocytes. Zhao et al. [27] demonstrated in cultured murine adipocytes that ERK activation induced serine phosphorylation of both STAT1 and p65. This in turn mediated the additive effects of IFNγ and TNF-*α* on NGAL expression. These studies suggest that NGAL plays an important role in adipocyte metabolism and inflammation. Chronic low-grade inflammation contributes to the occurrence and development of gestational diabetes. Lou et al. [12] demonstrated that NGAL and TNF-*α* mRNA and protein expression levels in SAT were significantly higher in GDM overweight women than in women with normal weight. It was noted that NGAL expression levels in adipose tissues were associated with insulin resistance in obese patients with GDM.

It was reported that NGAL levels were higher in pregnant women, notably when the prepregnancy body mass index was higher than >25 kg/m^2^ and that they correlated with markers of insulin resistance [28]. NGAL is expressed in trophoblast cells and its gene expression is upregulated in cytotrophoblast via stimulation by TNF-*α* [24]. Kobara et al. [29] suggested that the extravillous trophoblasts (EVT), cytotrophoblasts (CT), and decidua were the main sources responsible for the elevated plasma NGAL levels in pregnant women. Thus, we speculated that the elevated levels of NGAL in maternal circulation were mainly derived from adipose and placental tissues.

In addition, other organs can also produce NGAL. Haneda et al. [30] investigated the expression levels of NGAL in the gravid endometrium of mares, and found that endometrial NGAL could play a role in the transfer of small molecules from the mother to the fetus. A previous study demonstrated that NGAL-mediated LPS-induced inflammation in pregnant mice [31]. Moreover, Cao et al. [13] reported higher levels of NGAL protein in placental tissues of patients with preeclampsia, compared with those noted in normal pregnancies. Therefore, we hypothesized that NGAL in the placenta may play a role in fetal development.

It is well known that the placenta is an endocrine organ, which secretes various molecules to maintain normal physiological function during pregnancy [32, 33]. In vitro studies demonstrated that placental cells exert significant metabolic changes in response to high glucose, including fatty acid *β*-oxidation, phospholipid metabolism, and phosphatidylinositol phosphate signaling [34]. The umbilical cord is the link between the mother and the fetus and plays an important role in fetal waste metabolism and material exchange with the mother. Sun et al. [35] demonstrated that umbilical cord-derived mesenchymal stem cells (UC-MSCs) could attenuate insulin resistance due to their anti-inflammatory activity.

To the best of our knowledge, this is the first report that investigated the expression levels of NGAL mRNA and protein in terms of GDM placentas and umbilical cord. The present study indicated that the expression levels of NGAL and TNF-*α* mRNA and protein were both markedly higher in GDM women than those in the control group both in placental and cord samples. Moreover, maternal serum NGAL levels positively correlated with placental NGAL mRNA and protein expression levels in the GDM group. These results suggest that the increased expression of NGAL in the placenta may be responsible for its high maternal circulating levels in women with GDM, thus affecting the degree of insulin resistance. In addition, we found that maternal serum NGAL levels positively correlated with cord blood NGAL levels both in control and GDM subjects. Thus, we proposed that an association existed between NGAL levels in the maternal and the fetal circulation. Finally, maternal serum NGAL levels exhibited a positive correlation with neonatal birth weight in women with GDM and an increased expression of NGAL levels in the GDM group was noted both in cord blood and cord tissues, suggesting a possible association between NGAL and fetal development. However, the underlying mechanism requires further investigation.

There are some limitations in the study. Firstly, since NGAL is a factor associated with obesity, measuring NGAL levels in overweight pregnant women would be of considerable interest. Due to the small sample size, the conclusions cannot be extended to large human populations. Secondly, we did not investigate the NGAL expression levels following delivery. Thirdly, due to the small sample size, the study was not included a group with those treated with insulin. Therefore, the NGAL expression after delivery and in GDM patients treated with insulin should be investigated in future studies.

In conclusion, the expression levels of NGAL in placental tissues were markedly higher in GDM than in normal pregnancies. The oversecretion of NGAL from the placental tissues in women with GDM probably contributes to the increased serum NGAL concentrations and may further induce insulin resistance. The increased levels of NGAL in the placenta may play a role in fetal development. However, the exact role of NGAL in the pathogenesis of GDM and fetal development remains to be determined.

## Figures and Tables

**Table 1:** Clinical characteristics of normal pregnant controls and women with GDM.

	GDM (*n* = 49)	Control (*n* = 39)	*P* value
Age (years)	32.47 ± 4.68	31.77 ± 4.96	0.497
Gestational age (week)	39.14 ± 0.62	39.00 ± 0.46	0.275
Parity	1 (1, 1)	1 (0, 1)	0.346
Prepregnancy BMI (kg/m^2^)	23.15 ± 3.06	22.69 ± 3.00	0.488
Current BMI (kg/m^2^)	28.57 ± 2.96	29.19 ± 3.86	0.402
FPG1 (mmol/L)	5.00 ± 0.47	4.47 ± 0.34	<0.01
1 hPG (mmol/L)	10.07 ± 1.38	7.16 ± 1.39	<0.01
2 hPG (mmol/L)	8.04 ± 1.32	6.34 ± 0.89	<0.01
FPG2 (mmol/L)	4.92 ± 0.61	4.50 ± 0.40	<0.01
FINS (*μ*U/mL)	12.81 ± 4.62	10.72 ± 3.46	0.023
HOMA-IR	2.80 ± 1.11	2.15 ± 0.73	0.003
TC (mmol/L)	5.87 ± 0.99	6.28 ± 0.97	0.058
TG (mmol/L)	3.30 ± 1.36	2.63 ± 0.75	0.006
HDL-C (mmol/L)	1.76 ± 0.30	1.89 ± 0.27	0.041
LDL-C (mmol/L)	3.38 ± 0.73	3.61 ± 0.66	0.136
VLDL-C (mmol/L)	0.74 ± 0.27	0.78 ± 0.28	0.558
Birth weight (g)	3598.89 ± 430.05	3448.72 ± 366.97	0.092

Annotation: BMI: body mass index; FPG1: fasting plasma glucose in the second trimester; 1 hPG: 1 h plasma glucose after glucose loading; 2 hPG: 2 h plasma glucose after glucose loading; FPG2: fasting plasma glucose in the third trimester; FINS: fasting insulin level; HOMA-IR: HOMA-insulin resistance index; TC: total cholesterol; TG: triglycerol; HDL-C: high-density lipoprotein cholesterol; LDL-C: low-density lipoprotein cholesterol; VLDL-C: very low-density lipoprotein cholesterol. Data are mean ± SD and medians (25–75%); *P* values statistically evaluated as *P* > 0.05 insignificant and *P* < 0.05 significant.

**Table 2:** Relationship of maternal NGAL levels with clinical and demographic characteristics in the gestational diabetes mellitus (GDM) and normal glucose tolerance (control) groups.

	GDM (*n* = 49)		Control (*n* = 39)		All (*n* = 88)	
	*r*	*P*	*r*	*P*	*r*	*P*
Prepregnancy BMI	0.184	0.205	0.079	0.634	0.164	0.127
Current BMI	−0.083	0.572	0.073	0.661	0.014	0.900
FPG1	0.369	0.014	−0.009	0.956	0.334	0.002
1 hPG	0.012	0.943	−0.028	0.874	0.238	0.042
2 hPG	0.044	0.782	0.193	0.274	0.253	0.028
FPG2	0.390	0.008	0.461	0.003	0.486	<0.001
FINS	0.695	<0.001	0.358	0.025	0.559	<0.001
HOMA-IR	0.711	<0.001	0.479	0.002	0.624	<0.001
TC	0.179	0.240	0.019	0.908	0.066	0.550
TG	0.091	0.553	0.140	0.395	0.267	0.014
HDL-C	0.073	0.632	−0.035	0.830	−0.017	0.878
LDL-C	0.125	0.415	−0.008	0.962	0.097	0.381
VLDL-C	−0.112	0.463	−0.037	0.824	−0.041	0.715
Birth weight	0.363	0.014	0.239	0.142	0.350	0.001

Annotation: NGAL: neutrophil gelatinase-associated lipocalin; BMI: body mass index; FPG1: fasting plasma glucose in the second trimester; 1 hPG: 1 h plasma glucose after glucose loading; 2 hPG: 2 h plasma glucose after glucose loading; FPG2: fasting plasma glucose in the third trimester; FINS: fasting insulin level; HOMA-IR: HOMA-insulin resistance index; TC: total cholesterol; TG: triglycerol; HDL-C: high-density lipoprotein cholesterol; LDL-C: low-density lipoprotein cholesterol; VLDL-C: very low-density lipoprotein cholesterol; *r*: Pearson's correlation coefficient or Spearman's correlation coefficient; *P* values statistically evaluated as *P* > 0.05 insignificant and *P* < 0.05 significant.

**Table 3:** Relationship of maternal NGAL level with TNF-*α* and placental NGAL expression in the gestational diabetes mellitus (GDM) and normal glucose tolerance (control) groups.

	GDM (*n* = 49)		Control (*n* = 39)		All (*n* = 88)	
	*r*	*P*	*r*	*P*	*r*	*P*
Cord blood NGAL	0.349	0.014	0.399	0.012	0.490	<0.001
Maternal blood TNF-*α*	0.311	0.029	−0.065	0.696	0.248	0.020
NGAL mRNA	0.848	0.008	0.196	0.642	0.740	0.001
NGAL protein	0.636	0.011	0.246	0.377	0.600	<0.001

Annotation: NGAL: neutrophil gelatinase-associated lipocalin; TNF-*α*: tumor necrosis factor-*α*; *r*: Pearson's correlation coefficient or Spearman's correlation coefficient; *P* values statistically evaluated as *P* > 0.05 insignificant and *P* < 0.05 significant.

## Data Availability

The data used to support the findings of this study are included within the article.
